# Sensitive and selective method for the analysis of menthol from pharmaceutical products by RP-HPLC with refractive index detector

**DOI:** 10.4103/0975-7406.72141

**Published:** 2010

**Authors:** K. A. Shaikh, Sachin D. Patil

**Affiliations:** P. G. Department of Chemistry, Sir Sayyed College, P.B. No. 89, Roshan Gate, Aurangabad, M.S-431 001; India; 1 P.G. Department of Chemistry, Yeshwant Mahavidyalaya, Nanded-431 602, M.S., India

**Keywords:** Menthol, method development, method validation, refractive index detector, reversed phase HPLC

## Abstract

**Objective:**

Liquid chromatography with refractive index (RI) detection has been found to be very useful for the determination of menthol from pharmaceutical products. A simple and rapid HPLC method has been developed for this purpose compared to conventional GC methods, requiring no special sample pretreatment for the determination of menthol from pharmaceutical products.

**Materials and Methods:**

A chromatographic separation was achieved on a Inertsil ODS 3V (4.6mm×250mm, 5*μ*m) column using water : methanol (30:70 v/v) as a mobile phase, at a flow rate of 1.0 ml/min.

**Results:**

Method was validated as per ICH guidelines for various parameters such as precision, linearity, accuracy, solution stability, robustness, limit of detection and quantification. Results were found to be within acceptable limits.

**Conclusion:**

The method has been successfully applied for the quantification of menthol from syrup formulations. The developed method can be conveniently used by the quality control department to determine assay of menthol from pharmaceutical preparations.

Menthol is an organic compound made synthetically or obtained from peppermint or other mint oils [Figure 1]. Menthol has local anesthetic and counterirritant qualities, and it is widely used to relieve minor throat irritation in pharmaceutical products. Moreover, it is widely used in foods, beverages, cigarettes, toothpaste, and food flavor for its particularly refreshing taste. Although both l-menthol and dl-menthol have been used, main form of menthol occurring in nature is (–)-menthol. A variety of methods has been established for the analysis of menthol, including colorimetry,[1,2] gas chromatography,[3–8] HPLC with fluorescence-labeling reagents,[9,10] polarized photometric detector,[11] normal-phase HPLC with refractive index detector for separation of isomers,[12] and indirect photometry.[13] All these methods have many disadvantages, like low sensitivity except GC and HPLC using fluorescence-labeling reagents and lack of applicability for actual samples like syrup or tablet formulations because of the lengthy and difficult sample preparation. The thorough literature survey revealed that none of the most recognized pharmacopoeias or any journals includes this drug for the determination of menthol by RP-HPLC using refractive index detector. So it is felt essential to develop a liquid chromatographic procedure which will serve a specific, accurate, sensitive, and stability-indicating RP-HPLC method for the determination of menthol. The objective of our work was to establish a simple and rapid analysis of menthol in pharmaceutical products by HPLC using a refractive index detector to facilitate the specific detection of optically active menthol with no chromophores.

**Figure 1:**
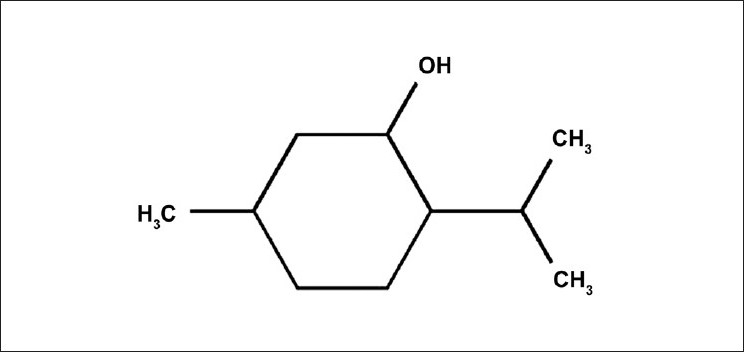
Structure of menthol

## Materials and Methods

Menthol (99.73%) was obtained as a gift sample from FDC Ltd., India. Methanol (HPLC Grade) was purchased from E. Merck (India) Ltd., Worli, Mumbai, India. The 0.45-*μ*m nylon filters were purchased from Advanced Micro Devices Pvt. Ltd., Chandigad, India. Milli Q water was used throughout the experiment. Cough and cold relief syrup samples were purchased from Indian market, containing menthol 2.5 mg per 5 ml.

Analysis was performed on a chromatographic system of Waters 2695 separation module (USA) equipped with an auto injector, Waters 2414 refractive index detector. A chromatographic separation was achieved on an inertsil ODS 3V (4.6 mm×250 mm, 5 *μ*m) column using water:methanol (30:70 v/v) as a mobile phase. Data acquisition was made with Empower software. Injection volume used was 100 *μ*l throughout the experiment.

### Standard solutions and calibration graphs

Diluent preparation – Used mixture of water and methanol (20:80 v/v).

Standard stock solution of menthol (0.5 mg/ml) was prepared in diluent. From this stock, standard solution was prepared by diluting 5 ml of this stock solution to 50 ml with diluent. To study the linearity range of menthol, serial dilutions were made by diluting 3, 4, 5, 6, 7 ml of stock solution in to 50-ml volumetric flask, made up the dilution with diluent and mixed.

### Sample preparation

Weight per ml (density) of the syrup sample was determined using pychnometer. Syrup sample equivalent to 2.5 mg of menthol was accurately weighed into 50-ml volumetric flasks; 5 ml of water was added and mixed. Further added 20 ml of methanol and the volumetric flasks were sonicated for 5 min with intermediate shaking and solutions were then made up to the volume with methanol and mixed. The solution was filtered through 0.45-*μ*m nylon filter.

### Method validation

The HPLC method was validated in terms of specificity, precision, accuracy, linearity LOD/LOQ, and solution stability according to ICH guidelines.[14] Assay method precision was determined using nine independent test solutions. The intermediate precision of the assay method was also evaluated using different analyst on three different days. The accuracy of the assay method was evaluated with the recovery of the standards from excipients. Three different quantities (low, medium, and high) of the authentic standards were added to the placebo. The mixtures were extracted as described in sample preparation, and were analyzed using the developed HPLC method. Linearity test solutions were prepared as described in Standard Solutions and Calibration Graphs. The LOD and LOQ for analytes were estimated by injecting a series of dilute solutions with known concentration. The solution stability study was carried out by injecting standard and sample solutions at different interval of time. To determine the robustness of the method, the final experimental conditions were purposely altered and the results were examined. The flow rate was varied by ±0.1 ml/min. The percentage of organic modifier was varied by ±5%. Column oven temperature was varied by ±5°C from ambient temperature.

## Results and Discussion

### Optimization of the chromatographic conditions

Conventional GC methods are helpful for the analysis of menthol from syrup formulations, but it requires tedious sample preparation like solvent extraction or time-consuming headspace GC. Our aim was to develop robust and reproducible HPLC method for the determination of menthol from syrup formulations. Due to lack of chromophore in the structure of menthol, it shows very limited absorbance in the ultraviolet region. So it is very difficult to analyze menthol with HPLC consisting of UV detector. So that we have decided to use refractive index detector as it is useful for components with limited or no UV absorption. Initially number of stationary phases like Zorbax SB-CN, Zorbax SB-C8, Inertsil ODS 3V, and methanol as mobile phase were used to separate menthol from excipient and other active ingredients in syrup formulation. From this inertsil ODS 3V (250 × 4.6) mm, 5 *μ* column is giving more retention time for menthol than other columns. Further 10% water is added in mobile phase along with methanol to increase retention of menthol and it is observed that menthol is eluted at 5 min with good peak shape. But this much retention is not sufficient as other ingredients in the formulation was interfering at the retention time of menthol. So that water composition is increased to 30% in mobile phase and menthol was eluted at 17 min separating from all other peaks. Column oven temperature and detector temperature set was 35°C to increase the peak sharpness. As menthol concentration is very less in syrup formulations, 100 *μ*l injection volume and 128 as detector sensitivity was selected to increase peak response.

### Validation of method

#### Specificity

**Figure 2:**
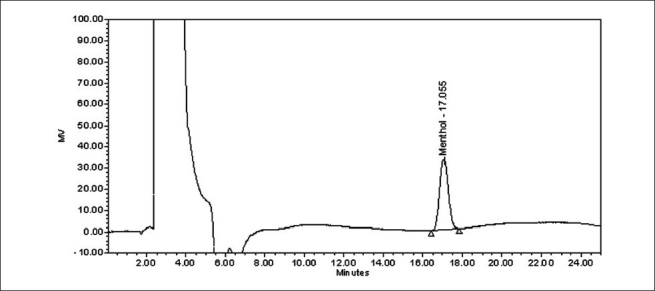
A chromatogram of menthol standard solution

**Figure 3:**
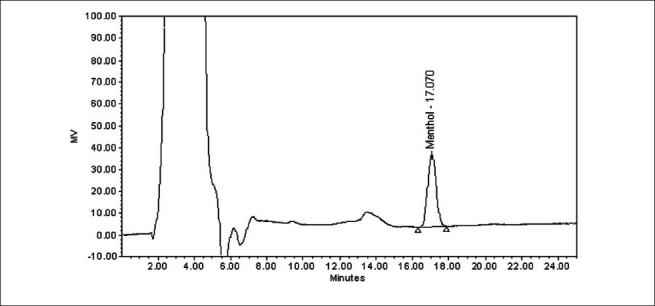
A typical chromatogram of syrup sample solution containing menthol 2.5 mg/5ml

The specificity of the HPLC method is illustrated in [Figures 2 and 3] where complete separation of menthol was noticed in the presence of cough syrup excipients. In addition, there was not any interference at the retention time of menthol in the chromatogram of placebo solution. Forced degradation studies were also performed on syrup samples to provide an indication of the stability indicating property and specificity of the proposed method. The stress conditions employed for degradation study includes light (1.2 million lux h/m^2^/22 h), heat (80°C/24 h), humidity (40°C/75% RH/7 days), acid hydrolysis (1N HCl/5 ml/3 h), base hydrolysis (1N NaOH/5 ml/3 h), and peroxide degradation (5% w/v H_2_O_2_/5 ml/3 h). About 2-5% degradation was observed in forced degradation studies. There was no interfering degradation peaks observed after 8 min in all the degradation conditions. This is due to higher organic content in mobile phase, which eluted all degradents earlier.

#### Accuracy

Accuracy of the method was calculated by recovery studies at three levels by standard addition method [Table 1]. The mean percentage recovery obtained was 100.4%.

**Table 1:** Results of the recovery analysis of menthol

Compound	Wt Spiked (mg)	Wt recovered (mg)	Recovery (%)	RSD (%) *n*=3
Menthol	1.500	1.504	100.3	0.71
	2.499	2.518	100.8	0.58
	3.499	3.503	100.1	0.61

RSD, relative standard deviation; Wt, weight.

#### Precision

The precision of an analytical procedure expresses the closeness of agreement between a series of measurements obtained from multiple sampling of the same homogeneous sample under the prescribed conditions. The system precision is a measure of the method variability that can be expected for a given analyst performing the analysis and was determined by performing five replicate analyses of the same working solution. The relative standard deviation (RSD) obtained for menthol was 0.40% [Table 2].

**Table 2:** System suitability parameters

Parameters	Menthol
Theoretical plates^a^	7500
Peak symmetry^a^	1.10
% RSD	0.40
Peak area	910220

**Retention time**	**17.055**

a USP–NF 29 section 621, pp. 2135.

The intra- and interday variability or precision data are summarized in Table 3. The intraday precision of the developed LC method was determined by preparing the standard solution in nine determinations with three concentrations and three replicate each. The RSD of the results was used to evaluate the method precision. The interday precision was also determined by preparing standard solution in triplicate per day for consecutive 3 days. The results indicated the good precision of the developed method [Table 3].

**Table 3:** Intra- and interday assay precision data (*n*=9)

Actual concentration	Measured concentration (*μ*g/ml), RSD (%)	
	Intraday	Interday
Menthol (μg/ml)		
30	30.14, 0.38	30.23, 0.78
50	50.44, 0.64	50.71, 0.52
70	70.11, 0.18	70.34, 1.25

Data expressed as mean for “measured concentration” values.

#### Linearity

**Figure 4:**
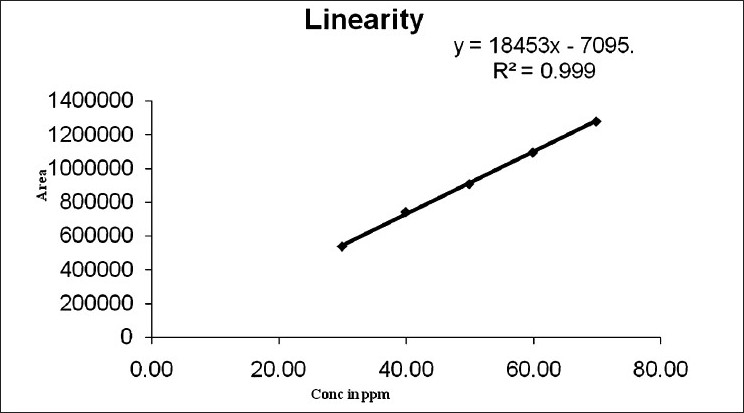
Linearity curve for menthol

Linearity was determined in the range of 30–70 *μ*g/ml for menthol. The correlation coefficient (r) observed was 0.999. Typically, the regression equation for the calibration curve was found to be 18452.7× -7095.5 [Figure 4].

#### Limit of detection (LOD) and limit of quantitation (LOQ)

LOD and LOQ of menthol were determined by calibration curve method.[13] Menthol standard solution was prepared in the range of 1.0–20 *μ*g/ml and injected in triplicate. Average peak area of three analyses was plotted against concentration. LOD and LOQ were calculated by using the following equations:

$$\mathrm{LOD}\;=\;\frac{3.3\;\times \;\mathrm{Syx}}{b};$$

$$\mathrm{LOQ}\;=\;\frac{10.0\;\times \;\mathrm{Syx}}{b}$$

where Syx is the residual variance due to regression; b is the slope.

LOD and LOQ for menthol were found to be 0.76 and 2.31 *μ*g/ml, respectively.

### Solution stability

To demonstrate the stability of standard solutions and syrup sample solutions during analysis, both solutions were analyzed over a period of 24 h while being stored at room temperature. The results showed that the retention times and peak areas of the drugs remained almost unchanged and no significant degradation was observed during this period, suggesting that both solutions were stable for at least 24 h, which was sufficient for the whole analytical process.

### Robustness

The robustness of an analytical procedure is a measure of its capacity to remain unaffected by small, but deliberate variations in method parameters and provides an indication of its reliability during normal usage.

To evaluate the robustness of the developed method, deliberate variations were made in the method parameters such as change in the flow rate, percentage of methanol in the mobile phase and column oven temperature. The standard solution was injected into the LC injector six times under different chromatographic conditions. The robustness of the method was evaluated by determining the effect of the modified parameters on retention time, tailing factor, area, and percentage of content. The degree of reproducibility of the results obtained as a result of small deliberate variations in the method parameters has proven that the method is robust [Table 4].

**Table 4:** Results of robustness study

Factor	Level	Mean % assay (*n*=3)	% RSD of results
Flow rate (ml/min)	0.9	100.5	0.77
	1.1	101.4	0.23
Column oven temperature (°C)	30	99.3	0.46
	40	100.7	0.92
% of methanol	65	100.2	0.89
	75	101.4	1.08

## Conclusion

An efficient RP-HPLC method compared to available HPLC methods for the quantification of menthol in drug product was developed and validated. The method is very simple and specific as all peaks were well separated from its excipient peaks with total runtime of 25 min, which makes it especially suitable for routine quality control analysis work. A comparison of menthol analysis by RP-HPLC method using refractive index detector with another methods is recorded in Table 5.

**Table 5:** Comparison of menthol analysis by RI detector with other methods

Method	Conclusion
Gas chromatography with flame ionization detector	Sample pretreatment required like extraction in volatile solvents, also low in accuracy.
HPLC using fluorescencelabeling reagents	Extraction and derivatization is required for sample.
HPLC using polarized photometric detection	Menthol peak is not well separated from placebo peaks. Also peak shape is not good as compared to present method with RI detector. Method specificity is not demonstrated.
HPLC using UV-detector	Low sensitivity as UV absorbance is very low.
RP-HPLC using RI-detector	Sample preparation is simple. Peak separation, peak shape, and sensitivity are better compared to other methods in literature. Method is fully validated as per ICH guidelines and can be used for routine quality control analysis.

## References

[1] Yurova NG, Popov DM, Denisova TA (1981). Photometric determination of menthol. Farmatsiya (Moscow).

[2] Safronova TO, Popov DM, Shevchenko TG (1982). Photocolorimetric determination of menthol in oil and tincture of peppermint. Pharm Chem J.

[3] Ligor M, Buszewski B (1999). Determination of menthol and menthone in food and pharmaceutical products by solid-phase microextraction-gas chromatography. J Chromatogr A.

[4] Gonzalez-Penas E, Lopez-Alvarez M, Martinez de Narvajas F, Ursua A (2000). Simultaneous GC Determination of Turpentine, Camphor, Menthol and Methyl Salicylate in a Topical Analgesic Formulation (Dologex). Chromatographia.

[5] Golubitskii GB, Dyuldina VV, Basova EM, Ivanov VM, Budko EV (2008). Chromatographic analysis of Passifit multicomponent pharmaceutical syrup. J Anal Chem.

[6] (2009). U.S. Pharmacopeia National Formulary, USP 32 NF27, US Pharmacopeial Convention, Rockville, MD.

[7] Li M, Nelson DL, Sporns P (1993). Determination of menthol in honey by gas chromatography. J AOAC Int.

[8] Zhang T, Wu JY, Tao JS, Zhang YF, Wu Y, He J (2007). Determination of menthol and caffeic acid in bohe dispensing granules. Chin Pharm J.

[9] Tutaya Y, Data Y, Kohashi K (1991). (2-Phthalimidyl) benzoyl Azides as Fluorescence Labeling Reagents for Alcohols in High Performance Liquid Chromatography. Anal Sci.

[10] Lin YT, Wu HL, Kou HS, Wu SM, Chen SH (2005). Enantiomeric analysis of (+)-menthol and (-)-menthol by fluorogenic derivatization and liquid chromatography. J Chromatogr A.

[11] Hamasaki K, Kato K, Watanabe T, Yoshimura Y, Nakazawa H, Yamamoto A (1998). Determination of l-menthol in pharmaceutical products by high performance liquid chromatography with polarized photometric detection. J Pharm Biomed Anal.

[12] Haut SA, Core MT (1981). Separation of Menthol Isomers by Normal Phase High Performance Liquid Chromatography. J Liq Chromatogr.

[13] Parkin JE (1984). High-performance liquid chromatographic assay of menthol using indirect photometric detection. J Chromatogr.

[14] (1996). ICH-Q2B Validation of Analytical Procedures: Methodology. International Conference on Harmonization of Technical Requirements for Registration of Pharmaceuticals for Human Use, Geneva, Switzerland.

